# Inuit Women’s Voices from Nunavut, Canada: Informing Perinatal Support Needs and Priorities

**DOI:** 10.3390/healthcare14152239

**Published:** 2026-07-23

**Authors:** Joanna Galasso, Mary Ann Forbes, Robyn Long, Nadine Alareak, Rosanna Amarudjuak, Gail Baikie, Judy Clark, Patricia (Patti) Johnston

**Affiliations:** 1 Faculty of Social Work, University of Calgary, 2500 University Drive NW, Calgary, AB T2N 1N7, Canada; joanna.galasso@ucalgary.ca (J.G.); maryann.forbes@ucalgary.ca (M.A.F.); robyn@arcticwellness.ca (R.L.); nadinealareak@hotmail.com (N.A.); ramarudjuak@gmail.com (R.A.); 2 Keeping the Children Home, University of Calgary, Calgary, AB T2N 1N4, Canada; gail.baikie@dal.ca (G.B.); judy.clark@umanitoba.ca (J.C.); 3 School of Social Work, Dalhousie University, 3201-1459 LeMarchant Street, Halifax, NS B3H 4R2, Canada; 4 Rady Faculty of Health Sciences, Ongomiizwin–Indigenous Institute of Health and Healing, University of Manitoba, 770 Bannatyne Avenue, Winnipeg, MB R3W 0W3, Canada

**Keywords:** Inuit, women’s health, perinatal care, sexual and reproductive health, medical evacuation for birth, equity, Nunavut, remote healthcare

## Abstract

**Highlights:**

**What are the main findings?**

- Evacuation-based childbirth policy in Nunavut, Canada, undermines Inuit women’s autonomy, limits informed decision-making, reinforces unequal power relations, and disrupts culturally grounded systems of care and support.
- Structural inequities constrain Inuit women’s ability to exercise their sexual and reproductive health and rights.
- Kivallirmiut women report a need for culturally relevant perinatal information and support that is grounded in Inuit Elders’ knowledge and peer networks, revealing important gaps in the design and delivery of existing perinatal services in Nunavut.

**What are the implications of the main findings?**

- Current perinatal care models remain rooted in colonial assumptions and are structurally misaligned with Inuit relational, cultural, and community realities in Nunavut, despite Inuit women expressing a clear desire for culturally grounded supports.
- These findings support the need for health system reform that integrates culturally and linguistically relevant perinatal education and care while strengthening Inuit-led, community-informed models across the perinatal continuum.

**Abstract:**

**Background/Objectives:** Evacuation policies for childbirth in Nunavut, Canada, continue to profoundly shape the lives of Inuit women, their families, and communities. The evacuation policy is known to disrupt kinship networks and contribute to the erosion of Inuit birthing knowledge. Addressing a gap in Inuit-led understandings of sexual and reproductive health priorities, this paper presents findings from research conducted in 2024 with Kivallirmiut women (‘People of’ in Inuktitut is miut; Kivallirmiut means people of the Kivalliq Region. Nunavummiut means people of Nunavut, and Arviarmiut means people of Arviat, Nunavut, Canada). The study took place in two communities in the Kivalliq Region of Nunavut. **Methods:** Employing a highly participatory, community-based Indigenous methodology, this study was underpinned by Inuit Qaujimajatuqangit, Indigenous feminist and postcolonial and decolonial theories, and principles of health equity. Data from a survey were generated to identify needs and priorities related to perinatal health and care. **Results:** Findings identify that Inuit women in these communities hold an interest in accessing perinatal information and support, with a strong desire for learning from Inuit Elders, as well as support via peer networks. Participants also emphasized a desire for more information concerning travelling for birth (medical evacuation), breastfeeding, and healing after birth. Different models for delivery of this information and perinatal support were also identified. **Conclusions:** Collectively, the priorities of Inuit women in this survey offer insights concerning a structural misalignment between existing perinatal care supports and services that rely on evacuation-based perinatal care and the needs and priorities within Kivalliq communities. This disconnection reveals enduring colonial assumptions embedded within current healthcare systems. The findings suggest a need for perinatal supports that centre culture, kinship, and relational forms of support.

## 1. Introduction

Within Nunavut, Canada’s northernmost territory, spanning approximately two million square kilometres and home to a predominantly Inuit population (85.8%) [1] living in small, remote communities, Inuit women are required to give birth in hospitals or in designated ‘approved centres’; obstetric services are unavailable in 24 of the 25 communities in the territory [2,3,4]. In this paper, ‘women’ and ‘mothers’ are used in accordance with the language most used in Nunavut and by participants in this study. We recognize that not all individuals who experience pregnancy and childbirth identify with these terms and may be better reflected by ‘those who birth.’ For almost 80% of women from the Kivalliq Region (seven communities on the mainland west of Hudson’s Bay and one of three regions in the territory), this requirement, known as medical evacuation, means travelling more than 1000 km to hospitals in Winnipeg, Manitoba, Canada, to give birth [5,6]. This policy, described as being implemented for clinical safety [7,8], is known to contribute to the displacement and disconnection of women from their families, communities, culture, language and land during one of life’s most significant events—thereby impacting cultural safety [6,7,8,9,10]. While clinical safety is critically important, existing research to date on this topic, and perinatal supports and services generally, has often focused largely on provider perspectives, healthcare and policy outcomes, and administrative data [11]. There is limited literature that explores the lived realities of Inuit women concerning cultural safety in perinatal care [6,11,12]. Few empirical studies have directly examined Inuit women’s perinatal priorities and experiences for support and their learning needs associated with birthing [13,14,15,16,17]. This leaves a gap in the understanding of formal and lay supports needed and desired by women in Nunavut.

To address this gap, the Inuit Perinatal Health Hub (IPHH) (the Inuit Perinatal Health Hub (IPHH), part of the Pan-Canadian Women’s Health Coalition and funded by the Canadian Institutes of Health Research (CIHR), was initiated in response to a request in 2019 from Inuit women in Arviat, Nunavut, Canada, to create perinatal resources. Those involved in the Hub include Inuit community leaders, community members, Elders, and youth in Nunavut, alongside Qallunaat (non-Inuit) and Inuit outside of Nunavut. The Hub is composed of those with lived experience, medical and health practitioners, and organizations and scholars from multiple institutions. All involved have dedicated themselves to Inuit self-determination and improved women’s health and wellness), and work under the direction and oversight of the Keeping the Children Home (KtCH) initiative (see https://www.keepingthechildrenhome.ca/ (accessed on 29 March 2026) to learn more). This research explores the lived experiences of Inuit women during the perinatal period, as well as their specific needs and priorities for education and support, with the intention of this learning being critical to the development of perinatal resources, and to maternal and child health. This article presents findings from a 2024 survey that was both co-developed and conducted with Inuit women from within the Kivalliq Region. The results highlight Inuit women’s perinatal priorities for support, and their self-identified learning needs associated with birthing. All stages of interpretation, including the identification of patterns and meaning making, were undertaken in close collaboration with Inuit women who are members of the research team and who hold oversight of the broader project. An in-depth, collective, and reflective analysis conducted with the larger research team and KtCH Governing Council to ensure comprehensive and lived analysis of the results is presented here. Our analysis emphasizes the need for Inuit-led, culturally grounded perinatal resources.

## 2. Background

The transfer of Inuit women from remote Nunavut communities to urban-located hospitals, rather than the investment in localized midwifery and birthing services in Nunavut over the past forty years, reflects significant structural inequities embedded in the Canadian healthcare system [6,18]. These inequities have been reported to undermine Inuit women’s rights to accessible, culturally safe, and self-determined perinatal care [6,8,10]. Long criticized, the evacuation policy for birthing in Nunavut can be understood as embedded within a colonial framework of medical care that has prioritized Western biomedical authority and state-defined standards concerning birthing safety, over and above Inuit knowledge, cultural ways of knowing and being, and autonomy [8,18]. The evacuation policy is also understood to have impacted community, social, and cultural networks of support and diminished opportunities for Inuit-led birthing efforts, which has displaced Inuit women from decision-making roles in their own and in their communities’ sexual and reproductive experiences [8,16,19].

Pauktuutit Inuit Women of Canada, the national Canadian organization representing Inuit women, have explained that evacuation for birth must be understood as being situated within broader structures of colonial governance and Inuit women’s ongoing efforts for sexual and reproductive justice and self-determination [15]. Centring the collective self-determination of Inuit in this study, therefore, serves to recognize that sexual and reproductive justice is “fundamentally a framework about power” [20] (p. 4). Individual self-determination can be understood as the rights of individuals to make decisions consistent with their values, beliefs, and perspectives [21,22]. Collective Inuit self-determination can be understood as the interconnectedness of individuals and the right of Inuit women to govern themselves and make autonomous sexual and reproductive healthcare decisions as a group [15,23]. Both individual and collective understandings of self-determination are critical to sexual and reproductive health and rights (SRHR) for Inuit in Canada.

Within Inuit contexts in Nunavut, SRHR can be understood as being grounded within a set of inherent, interrelated Indigenous rights. These rights are pre-existing, and fundamental; they include the rights to self-determination, land, culture, and resources, which are all recognized by the Charter of Rights and Freedoms, the Nunavut Agreement, the United Nations Declaration on the Rights of Indigenous Peoples (UNDRIP), and the Inuit Nunangat Policy, [15,24,25]. The Truth and Reconciliation Commission of Canada’s Calls to Action further emphasize the need for recognition and implementation of these rights within Canadian government health policy [26,27,28] (For example, the Truth and Reconciliation Commission of Canada: Calls to Action, #18: “We call upon the federal, provincial, territorial, and Aboriginal governments to acknowledge that the current state of Aboriginal health in Canada is a direct result of previous Canadian government policies, including residential schools, and to recognize and implement the health-care rights of Aboriginal people as identified in international law, constitutional law, and under the Treaties” [26]). Inuit rights are also based in unwritten Inuit maligait (laws) and piqujait (rules), which have always been known and observed by Inuit [29]. While occasionally varying, given location and individual and family preferences, birthing within Inuit contexts is known to have always involved families and/or community members in reproductive decision-making and the experience of birthing [29].

Both the inherent rights associated with birthing and understandings of Inuit self-determination can also be understood as being grounded within Inuit Qaujimajatuqangit (IQ; commonly understood as Inuit, Indigenous knowledge), which are often described as “the ways of the Inuit” (p. 35 [30]). This is because IQ encompasses understandings and principles of historical, ongoing, and emerging understandings of intergenerational knowledge, relational accountability, and land-based ways of knowing, among other things. IQ also emphasizes a nurturing of kinship and land-based relationships throughout the perinatal period [8,16,31]. As a result, the current evacuation policy, which requires Inuit to leave their lands, communities, and culture to give birth in hospitals, can be understood by Inuit as being in direct conflict with IQ. When taken together, a restoration of Inuit sexual and reproductive autonomy over birthing is both a matter of health equity and decolonization [8,26].

## 3. History of Birth Evacuation in Nunavut

A long-standing birth evacuation policy has required Indigenous women in rural and remote communities in Canada to travel to hospitals to give birth [5,9,18]. In Nunavut, this national policy that has been described as originating in the 1960s became more consistently applied in the 1970s [6,18]. Prior to this policy, however, childbirth in Nunavut was historically supported within families and communities; today, this support provided during birth is typically understood as midwifery [6,15,32]. Importantly, this support, which was provided most commonly by women to women through traditional Inuit midwifery approaches historically, was documented as adequate to ensure the health of women and infants [9]. Examples of this can be seen in archival documents dating back to the 1930s, such as that of a visiting medical doctor in Pangnirtung, Nunavut who wrote: “Many of the customs used by the Eskimo have been derived from experience in the Arctic for generations and have proven the most valuable under the prevailing circumstances… the women are quite apt midwives… [and their specific form of midwifery] to me was quite a good idea” (pp. 1–2 [33]). Over time, however, traditional Inuit midwifery, which was community-based, culturally responsive and highly suitable to the context of the Arctic, became increasingly reframed by southern medical authorities as medically risky, but also “as an influential way to assimilate and civilize First Nations into the colonial world” ((p. 332, [34]); [33,35]).

Notably, the increasing discourse concerning risk and framing Inuit birthing as risky, should be understood within the context of wider patterns of colonial governance, including the imposition of permanent settlements in Inuit Nunangat. In Canada at this time (and often still today), government agents assessed Indigenous peoples’ health and social wellness against middle-class, dominant cultural Westernized standards and assumptions [35]. This assessment contributed to the *Sixties Scoop*, during which Indigenous children were systematically apprehended from their families due to what were typically structural concerns, such as poverty and housing insecurity [36]. Such Western understandings were often lacked a recognition for context, structural conditions, and, most importantly, Inuit voice and body sovereignty. The discourse concerning risk also laid the groundwork for the broader medicalization of childbirth, wherein clinical intervention was posited as both necessary and self-evident in response to perceived obstetric risk [6,24,37]. This period marked a decisive transition from home-based childbirth, supported by family members and/or ikajurti (helpers), to medically supervised deliveries in federal nursing stations [9,38,39]. During this time and into the 1970s, many Inuit women continued to give birth locally, but increasingly, they gave birth with nurse practitioners and internationally trained midwives [32]. By the late 1970s, medical doctors assumed primary responsibility for obstetric care in Nunavut [32] as shifts in federal immigration policy led to the withdrawal of UK-trained midwives, further reshaping the maternity care landscape [39]. Where initially this rationale of medically-supported birthing, continued to result in community-based birthing, perspectives concerning medical oversight soon evolved to justify the evacuation of pregnant Inuit women to southern hospitals [32]. Expanding accessibility and profitability of air travel further contributed to a rise in medical evacuations, including in cases where they were not clinically indicated [39]. In the Kivalliq region (formerly known as the Keewatin district), this meant travel to Churchill, or even Winnipeg, more than 1000 km to the south [32,39]. These shifts enshrined the medical model within a redefined understanding of childbirth as a condition requiring urban-located, specialized, typically gendered, medical oversight [40]. These early evacuation practices were quickly normalized, and today they have been understood not simply as a clinical response, but as an extension of colonial surveillance and governance [38,39]. The result was the encouragement of the Inuit women to become passive recipients of a healthcare system that devalued local, culturally grounded expertise and perpetuated dependency on southern-based institutions and Western medical knowledge [34,41]. Thus, the evacuation policy functioned to fundamentally restructure Inuit birthing, but also, by extension, the policy served to rupture relationships, family life, mobility, and social and cultural systems of care, among other things.

## 4. Medical Travel for Birth Today

Although rooted in past colonial understandings and processes, evacuation for birth remains a routine feature of contemporary perinatal care in Nunavut, which still requires Inuit women to leave their communities during the later stages of pregnancy (typically at 36 to 38 weeks) to await delivery [16,39,42]. Inuit women have reported experiencing isolation and loneliness during this time of their pregnancy and birthing, due to separation from their other children, families, communities, Elders, and the social, cultural, and spiritual supports that traditionally surround pregnancy and childbirth [9,42,43]. The emotional toll and stress of being separated from familiar and trusted support networks can be compounded by experiences of impersonal and culturally insensitive socio-health care in urban hospitals, and feelings of being unsafe [7,17,43,44]. This stress can be intensified further by structural limitations, including the material and logistical challenges that can arise during evacuation. For example, when a spouse, partner, family member or friend, wishes to escort a pregnant person during the medical evacuation period, this typically requires arranging or assuming childcare and caregiving responsibilities, as well as absorbing income loss due to missed employment; costs that are disproportionately borne in contexts where economic opportunities are already limited and frequently tied to seasonal or hourly labour [6,10]. These challenges can be further intensified by inconsistent communication, administrative processes, and policy interpretation across jurisdictions, as well as limited awareness of escort eligibility criteria, all of which create additional barriers to accessing support during medical travel [10]). Further, in southern urban centres, such as Winnipeg, few Qallunaat (non-Inuit) healthcare workers hold an awareness of Inuit social and cultural traditions, values and approaches to care [27,45]. As a result, the birth experience for many Inuit can include feelings of confusion, powerlessness and alienation, factors that are known to impact women’s mental health [7,16,43,44].

For over 50 years, Inuit women were required to travel for birth alone [6]. The policy is often understood as being needed for institutional efficiency, cost containment, and biomedical risk management [43]. In 2017, policy change enabled pregnant Nunavummiut women to travel with one support person (termed a medical escort) [2,41]. Despite this change, evacuation continues to be perceived predominantly negatively by Nunavummiut [2,42,46] (briefly, from 2017 to 2026, the Inuit Child First Initiative enabled some Inuit women to bring both an escort and child/their children with them during evacuation [47,48]). This negative perception may be because the inclusion of a medical escort was presented as a move toward culturally sensitive care, but in practice, the policy instead reveals the persistence of structural inequities and colonial assumptions concerning birthing [39]. This is because accompaniment of one individual may be considered reasonable within a nuclear family model and treated as accommodation. However, a one-escort policy may also signal a distance between Inuit family- and community-based cultural approaches for birthing. The policy may also be better framed as a permission, as it can be both restricted and withdrawn, rather than provided as a fundamental right. The medical policy also narrowly defines and limits eligibility, and as a result, privileges a medically sanctioned form of support [2]. Thus, Inuit women’s rights, but also their priorities and needs, appear to remain structurally constrained. As a result, existing evacuation policies in Nunavut, such as this one, can reinforce unequal power relations and position cultural connection and relationships as an add-on rather than as a foundational principle of care [6,16].

The experiences of Inuit women who are evacuated for birth in Nunavut differ significantly from those of women who travel for maternity care in other remote and rural regions of southern Canada. Maternity-related travel in southern contexts is generally shaped by referral pathways and service distribution within familiar linguistic, cultural, and geographic environments. Such travel is often undertaken through a combination of personal preference and clinical referral and is typically supported by access to a broader range of healthcare, social, and familial supports. In contrast, birth evacuation for Inuit women, who are often young during pregnancy (Inuit have the youngest maternal age in Canada where almost one quarter, 24% were under the age of 20 years old at the time of giving birth in 2017) [49,50]), frequently involves prolonged separation from family, community, language, culture, and traditional support networks, resulting in a fundamentally different maternity care experience [43,51]. This experience can also mean navigating unfamiliar urban health, social and geographic systems, settings, and policies, sometimes for the first time or with limited prior experience in the urban environment [10,17,19]. This experience can be particularly impactful, given that the North is characterized by open tundra, immediate access to the land, and small, predominantly Inuit communities with strong kinship ties and cultural continuity, whereas Winnipeg, as an example, is an urban, densely built space, and it is governed by private land and fast pace, which can compound feelings of fear and isolation. Further, evacuation from Nunavut requires new mothers to navigate airports with newborns, including weather and other travel delays, and this experience of travel is not an exceptional circumstance, nor an option, but instead a routine, state-mandated feature of healthcare delivery for the vast majority of Inuit women [10,16,43,52].

## 5. Known Impacts of Evacuation for Birth

Evacuation for birth in Nunavut has been reported to compromise women’s health and weaken the cultural and social fabric of communities across generations [16]. Repeated disruption of community-based birthing has served to undermine intergenerational knowledge transmission and erode women’s traditional roles; roles that are understood as central to Inuit kinship, identity and social organization [16]. Birth evacuation from community can also diminish opportunities for collective care, storytelling and cultural learning during the perinatal period, processes that can underlie the formation of identity [10,16]. As a result, impacts of maternal evacuation can extend beyond the perinatal period and reverberate across generations as a form of structural and cultural loss [19,37].

For Inuit who experience colonial surveillance and intervention within Canadian child welfare systems (child protective services; CPS), evacuation to southern urban hospitals for childbirth can create heightened levels of stress [45,53]. This is because within hospital settings, Inuit women, like many Indigenous women in Canada, have reported encountering monitoring, scrutiny, and judgement [53,54]. Surveillance of Inuit women has been associated with biases towards Indigenous mothers as unfit caregivers [44,53,54,55]. Where surveillance may be understood as protection, healthcare, and necessary medical authority, such surveillance can also be understood as a form of sexual and reproductive governance over women’s bodies, lives, actions, decision-making, and family structures [10,19]. When surveillance is experienced alongside the structural challenges of evacuation, an intensification of stress and vulnerability in urban centres may further contribute to an undermining of reproductive sovereignty [45,53,54,55].

The inequities Nunavummiut women experience may contribute to poor birth outcomes for Inuit as well. Specifically, preterm birth rates (infants born prior to 37 weeks) among Inuit women between 2004 and 2006, of which stress during pregnancy is understood as a contributing factor, were almost twice that of the national average (11.4%; 95% CI: 9.7% to 13.1% for Inuit women vs. 6.7%; 95% CI: 6.6% to 6.9% for non-Inuit women) [50] (preterm birth rates have been reported at 16.5% for Inuit-occupied locations in Canada in 1990–2000 [56], 18.2% in the Baffin Region of Nunavut between 1998 and 2000 [57], and currently at 14.3% for all of Nunavut in 2024 [58].). Importantly, infant mortality in Nunavut have been increasing and recently were reported to reach a staggering 21.2 deaths per 1000 births in 2024—nearly five times the national rate of 4.6—also underscoring a profound and unacceptable inequity in early life outcomes for Inuit children [59]. These rates can be compared to the entire Northwest Territories between 1983 and 1985, which, at the time, included what is now Nunavut [60]. Infant mortality was reported at that time to be 28 deaths/1000 live births (and over 25 per 1000 total births in the 1970s) [32,61]. These rates indicate that despite the evacuation policy being in place for over 40 years, infant mortality has only moderately decreased in Nunavut. Further, during evacuation, medical interventions employed can often result in physical trauma and longer-term health challenges for both mothers and infants [16,61]. With such significant health disparities for Inuit in Nunavut, medical evacuation, which is often conceptualized as necessary to ensure equal access to healthcare for northerners compared to those in southern urban Canadian locations, does not result in equal health outcomes [6,10,19,45].

## 6. Methodology

Our research has been built from and remains grounded in IQ, often understood as Inuit knowledge (both traditional and contemporary understandings) or the “the past, present and future knowledge, experience and values of Inuit society” (p. 35 [30]). We underpin our work with understandings of Inuit ways of knowing and being to ensure our methodology centres current lived experience and can be “reconciled with what is important to Nunavummiut” (p. 36, [30]). IQ provides a foundation to understand Inuit women’s knowledge and lived realities concerning pregnancy and birth as a social, cultural, and relational experience. IQ principles also guide our interpretation of relevant literature and the data collected, and as a result, reveal how evacuation for birth can disrupt kinship, cultural continuity, and self-determination [62].

Postcolonial and decolonial theories contribute to a contextualization of this knowledge and experience within historical and current-day colonial power relations [63,64]. Indigenous feminist theory (IFT) further emphasizes the gendered dimensions of colonialism and caregiving, and the resistance and resurgence of Indigenous women [18,65,66,67,68]. IFT also provides a background of understanding for how women, their bodies, knowledge, and leadership are regulated, devalued, and undermined during pregnancy, birth, and caregiving [18,66,67,68]. The use of community-based participatory research enabled us to operationalize this theoretical framework, while emphasizing collaboration, reciprocity, shared authority, and reflexivity in knowledge production [69,70].

Beginning in 2019, Inuit women’s perinatal priorities and interests were discussed in both small and larger group meetings with women in Arviat, Nunavut. Repeatedly, we received a request for perinatal resources. Subsequent meetings over the next three years were held with the goal of coming to a better understanding what this request for perinatal resources entailed. The meetings resulted in the co-development of this research to be conducted with women in the Kivalliq Region in two communities. Our research team that conducted this research and engaged in data collection included two Inuit Elders and both Inuit and Qallunaat researchers (seven individuals in total). Data was collected in Arviat and Kangiqłiniq (Kangiqłiniq, also known as Rankin Inlet, is represented by several Inuktitut spellings from various dialects and orthographic variations which may evolve over time, including Kangiqłiniq, Kangiqliniq, Kangirliniq, and Kangiqtiniq, meaning deep bay/inlet). Both Arviat and Kangiqłiniq are considered the largest communities in the Kivalliq region, with estimated populations of 3170 and 3379, respectively [70]. The communities hold different experiences associated with evacuation for birthing, given that Kangiqłiniq hosted an Inuit maternity centre, where Inuit could give birth in the community from 1993 to 2020 [71,72]. This ability to give birth in Kangiqłiniq officially ceased in 2023 [71,72]. Many reasons are known to have contributed to the closing of the Kangiqłiniq maternity centre [71,72]. Inuktitut is the primary language spoken in both communities [73].

The methods employed in this study were developed to provide opportunity for women to reflect on their needs and priorities for perinatal support, the types of perinatal information considered most relevant to their past and present perinatal experiences, their preferred modes of perinatal information delivery (e.g., in-person, digital, print, etc.), and topics and issues they believed most important for both themselves and other Inuit women, families and youth. A short survey (approx. 10 min) was conducted in person to ensure accessibility. Surveys were recorded in English, but the option to complete them with Inuktitut-speaking research team members was made available. Recruitment to participate in this study was conducted over community radio, social media, and by way of word of mouth. Surveys were completed in a one-week period within the two communities. Participants met researchers in conference rooms, local grocery stores, and at women’s homes as desired. Participants received a $50 grocery store gift card prior to participating in the survey, which they were advised they could keep even if they chose at any point to stop participating, or to not participate at all. In Nunavut, all basic items for purchase at local grocery stores are substantially more expensive than in southern Canadian provinces. Given this difference, these honoraria provided here can be compared to a much smaller amount (e.g., $25) in urban locations. No one opted to stop participating during the survey, nor chose not to participate in the study upon learning about it.

The survey instrument itself was co-developed with Inuit women. It was also informed by collaboration with local organizations, perinatal practitioners, and community leaders. The survey was repeatedly reviewed to ensure the clarity, relevance, and alignment of what was learned through meetings and consultations. Organized into sections that included perinatal support and resources, women were asked to respond to questions regarding the perinatal supports they had previously received or required to identify perinatal educational topics they wished to learn about, and to offer other desires and interests associated with their past and present perinatal experiences. Those who expressed an interest in providing support to others were also asked how they preferred to assist others and why. The women could select from a predetermined list of options. Completed surveys were reviewed by the research team and manually entered in Microsoft Excel. Descriptive statistics were used to summarize demographics and survey responses. Closed-ended questions (quantitative results) and selected items for ‘other’ (qualitative responses) are presented here [16].

Countless team discussions and iterative comparisons of responses informed the analysis of the survey data. The analytic process was grounded in the recognition that knowledge is generated through multiple, equally valid ways of knowing and is not limited to rational–empirical approaches alone [74]. In this framing, experiential knowledge is treated as a legitimate and rigorous form of evidence that emerges through critical reflection, relational engagement, and sustained practice. This approach aligns with Indigenous epistemologies and methodologies, including IQ, that understand knowledge as contextual, relational, and co-created through lived experience, community, culture, and shared inquiry [75,76].

Accordingly, our research team engaged in what has been described in the international Indigenous literature as a herringbone stitch model of analysis, given the movement between stages of analysis [77]. This framework for understanding knowledge as iterative, relational, and co-constructed—as an Indigenous methodology, it emphasizes cyclical interpretation and ensures Indigenous women’s ways of knowing, being, and doing are the foundation for initiating analysis [77]. Rather than privileging detached observation, the team engaged reflexively with their own individual and collective cultural, academic, professional, and lived experiences, and together considered how this shaped the interpretation of findings. Knowledge was developed through ongoing dialogue, collective sense-making, and sustained attention to diverse perspectives, with particular emphasis on Inuit ways of knowing and the relationships that informed both the research process and its interpretation.

## 7. Results

A total of 83 surveys were completed within this study. Of these 83 surveys, 32 surveys were completed in Kangiqłiniq, and 51 surveys were completed in Arviat. Kangiqłiniq, which serves as a regional travel hub, included responses from women who reported that their home communities were Tikirarjuaq (also known as Whale Cove, population 528) and Salliq (also known as Coal Harbour, population 1128), both of which are neighbouring communities within the Kivalliq Region [70]. All those who participated in this study identified as female. Participants were invited to provide demographic information, including their age; however, completion of these items was voluntary and not required for participation. Among those who reported their age (n = 70), participants ranged from 17 to 74 years, with the majority being under 35 (66%) and over 50 years old (10%). Based on the ages provided and the research team’s reflections during data collection, most respondents appeared to be younger Inuit women, with a smaller number of Elders also participating. No additional demographic information was collected to maintain participant confidentiality and minimize survey burden. Three categories of questions were included: supports during pregnancy and after birth, educational interests, and supporting other women.

Findings are presented separately for Arviat and Kangiqłiniq to honour the distinct experiences and perspectives of participants within each community. Although both communities are located within the Kivalliq Region and in Nunavut, important differences in local context, service availability and accessibility, language, culture, and social and community experiences may shape perinatal needs and priorities. These community-specific factors are described in more detail and explored further in our Discussion below. Reporting findings separately enabled the identification of both common and community-specific priorities while avoiding assumptions of identical experiences, priorities, and needs.

**Figure 1:**
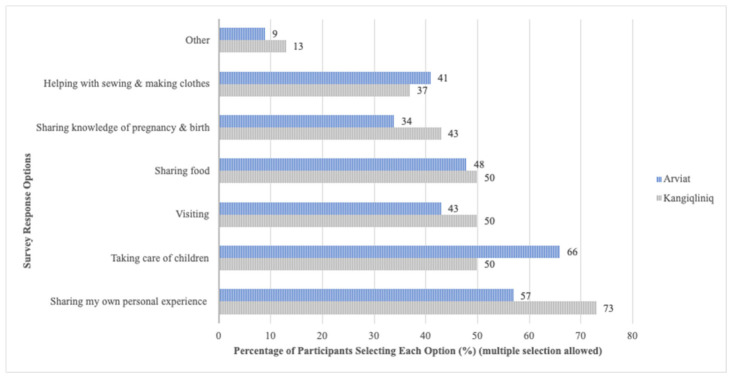
Participant-reported support needs and priorities during pregnancy and the postpartum period.

Across both communities, participants highlighted a need for accessible, culturally grounded and practical information during the postpartum period. Participants were asked, “What support did you need, or wish you had, during pregnancy and after you give birth?” Many participants identified the need for access to information related to travelling to Winnipeg for birth, as well as opportunities to participate in prenatal classes where they could engage with other mothers (see Figure 1) (note: for all questions, participants could select multiple response options; percentages represent the proportion selecting each option).

Participants described needing additional perinatal information and education. Gaps in information, particularly for younger women, were noted. This included specific medical information that could prepare them for giving birth, but also cultural and practical information.

In Arviat (n = 51), just over half of all participants (51%) indicated that information about travelling to Winnipeg to give birth was needed. Similarly, in Kangiqłiniq (n = 32), most participants (69%) also identified information about travelling to Winnipeg to give birth as needed. Despite the requirement for women to travel to give birth in Winnipeg, participants reported that little information was provided to prepare them for this experience. Where participants selected Other in this section of the survey, comments included needed perinatal supports specific to those who are deaf and hard of hearing (in 2000, it was estimated that almost 6% of the population in Nunavut was deaf or hard of hearing [67,78]), as well as basic needs to be met with increased finances (e.g., clothing). Importantly, this was the only component of the survey where those in Arviat selected this item more than those in Kangiqłiniq.

The limited information participants reported having access to was tracked beyond that of medical travel information to include perinatal education, cultural support and education, and practical information as well. Over half of participants (59%) in Kangiqłiniq reported that both prenatal classes (in-person) and support groups with other women are needed resources during the perinatal period. Some attended and received local prenatal supports in their communities, but commented on requiring more specific information (e.g., medical, practical, and cultural, etc.). Of all the participants in this study, nearly half (47%) of all participants stated that having a support group where they could have the ability to meet with other women was needed during pregnancy and following birth.

**Figure 2:**
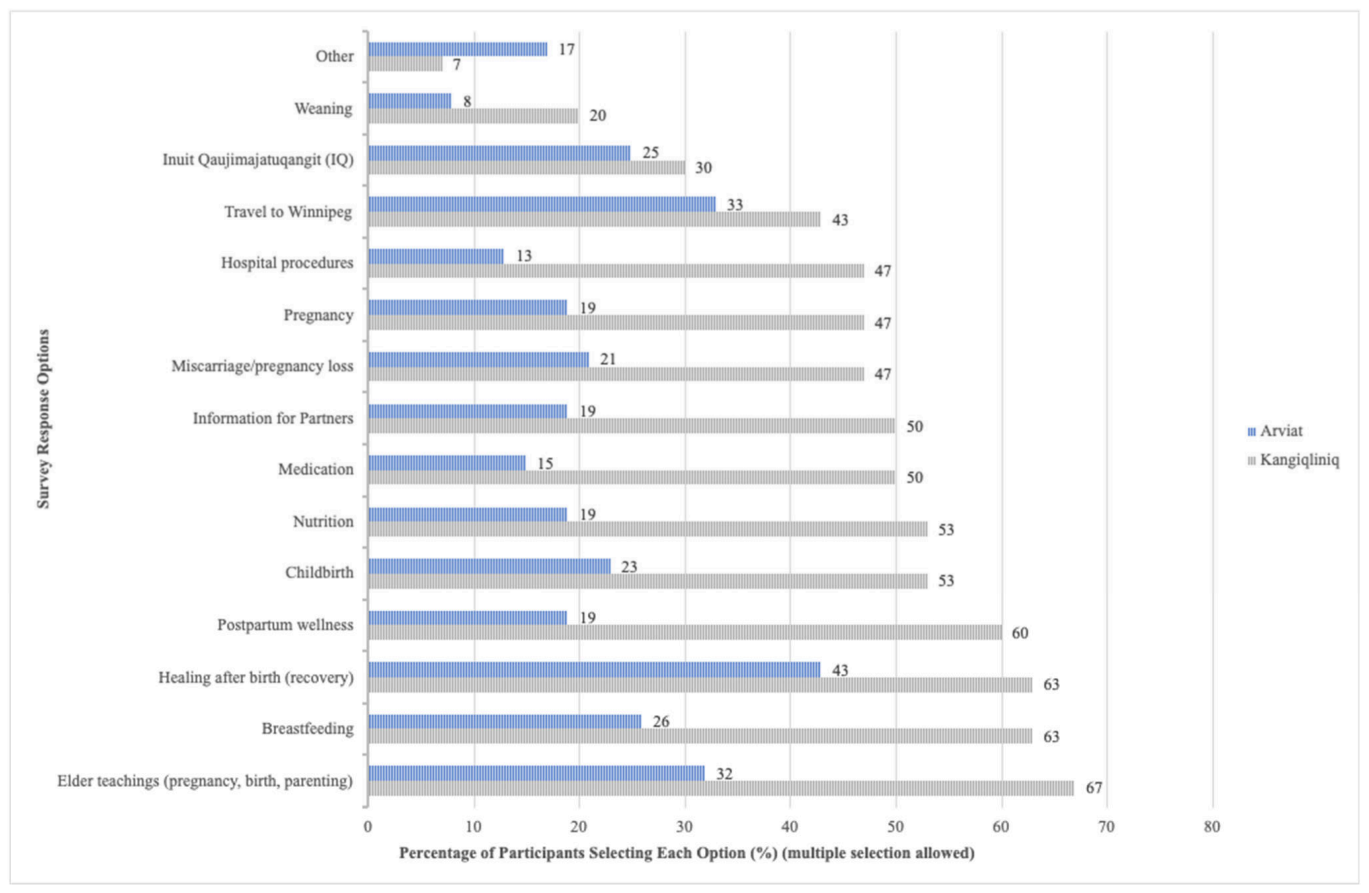
Participant-reported learning needs related to pregnancy, birth, and early parenthood.

In the second section of the survey, participants were asked about perinatal topics of interest and need. Specifically, they were asked: “What do you wish you knew about before you were pregnant, gave birth, or had a baby?” When asked these questions, participants indicated culturally specific knowledge and education to support their healing and wellness (see Figure 2).

In Kangiqłiniq (n = 32), 67% of the participants indicated an interest in learning from Elders and gaining more cultural understanding associated with pregnancy, birth, and parenting. The participants in this same community also noted the importance and desire to access perinatal information associated with healing after birth (63%), breastfeeding (63%), and postpartum wellness (60%). Importantly, these same items in Arviat (n = 51) were selected less often. Arviarmiut participants instead indicated a desire for information concerning healing after birth (43%), travel to Winnipeg (evacuation) (33%), and Elders’ teachings concerning pregnancy, birth, and parenting (32%), and breastfeeding (26%). Notably, across both communities, over one-quarter of the participants who engaged in this survey (27%) indicated a desire for perinatal resources and information for partners (e.g., fathers). Those who selected Other indicated a desire for information for their partners, which was more often reported in Arviat than in Kangiqłiniq. Importantly, group reflection and analysis of healing after birth (recovery) (63% in Kangiqłiniq and 43% in Arviat) and postpartum wellness (60% in Kangiqłiniq, 19% in Arviat) led to significant conversation and reflection among the research team. Insights from this conversation are described below in the Discussion.

**Figure 3:**
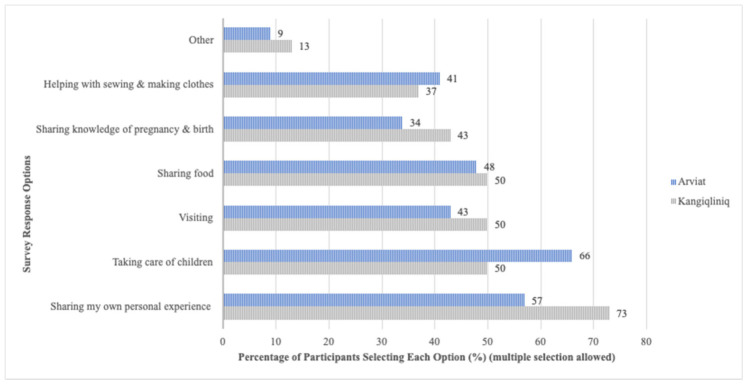
Preferred ways of supporting other women among participants.

Finally, given previous discussions and the co-development of the study, social support had previously emerged as a central theme. Here, again in the survey, 89% of women indicated they desired to help others during pregnancy and after birth. After noting an interest in supporting other women, participants were asked, “How do you like to best support other women?” Participants could select multiple response options; percentages represent the proportion of participants selecting each approach (see Figure 3).

The most common ways participants in Kangiqłiniq reported preference for supporting other women included sharing their own experiences of pregnancy and birth (73%) and taking care of children, sharing food, and visiting (going to visit women in their homes) (all 50%). In Arviat, participants selected taking care of children (66%) most often, and that they preferred to share their own personal experience with pregnancy and birth (57%) and sharing food (48%). Those who selected Other here indicated that they also preferred to support other women through helping with household tasks such as cleaning and snow removal (e.g., from steps and/or in front of someone’s home, etc.), hunting, and “being a friend.”

## 8. Discussion

Important themes that arose within our analysis of these findings include Cultural Guidance of Elders, Inuit need Inuit, and Structural Barriers and Inequities. The results from the surveys, relevant literature, and our analysis are discussed here.

### 8.1. Guidance of Elders

Our survey findings indicate that the research participants were interested in accessing information concerning cultural knowledge that Elders could offer during the perinatal period, including on topics of pregnancy, birth, and parenting. Participants also expressed a desire to learn more about IQ. The research team spent considerable time discussing the results and noted key aspects of understanding that can be gleaned. Notably, the higher reported interest in learning from Elders occurred in Kangiqłiniq, which, relative to Arviat, may reflect the differences in community structure, mobility, and access to extended kin networks. As a regional hub within the Kivalliq Region, Kangiqłiniq attracts individuals and families from surrounding communities for employment, education, and services. Research suggests that migration to regional centres can disrupt regular access to intergenerational support and cultural mentorship, increasing reliance on more formal or organized learning opportunities [79]. This potential migration from home communities to Kangiqłiniq may have resulted in geographic, social, and cultural separation from support networks for some participants, including extended family members and Elders, who often play a central role in the transmission of perinatal knowledge. This separation may have resulted in a greater interest in accessing learning from Elders. Importantly, the differences in results that exist between Arviat and Kangiqłiniq should not be interpreted as reflecting divergent cultural values, but rather as variations in access to knowledge shaped by patterns of settlement and social organization. The interest in learning from Elders across both communities underscores the enduring importance of Elders’ roles and knowledge in supporting women during the perinatal period.

In contrast, Arviat does not tend to have the same in-migration of Inuit from other communities as Kangiqłiniq. Many Arviarmiut may live in closer proximity to extended family members, including Elders. This could be why Arviat is often described as remaining closely connected to its traditional Inuit roots [80]. Having Elders in close physical proximity may facilitate ongoing, informal learning through daily interaction, observation, and shared caregiving. Such conditions may be reflected in the lower reported need for learning about IQ and knowledge possessed by Elders. In effect, this may mean that access to IQ and intergenerational knowledge in Arviat is simply embedded within everyday life for many of the participants and, as a result, fewer participants saw the need to access this learning.

Taken together, and highly consistent with the existing literature, these findings point to the important role Elders play in the provision of culturally relevant perinatal support, and the desire for intergenerational transmission of perinatal information [81,82]. Across health and social service sectors, Elders are widely recognized as critical to strengthening cultural safety, reinforcing Indigenous identity, and supporting resilience [79,82,83]. Integrating Elder-led education into perinatal care, therefore, aligns with decolonizing approaches to health by restoring Inuit authority over sexual and reproductive knowledge and decision-making, and by reaffirming Inuit epistemologies [82]. It also highlights the importance of grounding perinatal education within kinship and relationality. However, while Inuit participants in both communities seek perinatal supports that reflect Inuit culture, these supports may vary. Further research is needed to better understand the nuances in these needs within each specific community, and potentially how regionalization, migration, and access to extended kin networks may shape experiences and pathways to cultural knowledge and support.

### 8.2. Inuit Need Inuit (Collective Perinatal Care)

Within the Kivalliq region, participants indicated a desire to support one another alongside interest in IQ-based resources. An interest in activities of support, such as caring for others’ children, visiting, and sharing food, signal the enduring importance of collective care. Historically, and continuing today, responsibility for the wellbeing of the birthing parent and infant within Inuit contexts has been understood as communal, actively upheld by kin and community members, rather than ceded exclusively to a medical authority, and subsequently transferred to a nuclear family unit [9,10,39,84]. This suggests perinatal support needs and priorities in Nunavut must be shared and grounded in relationships between and among women, with family, and community.

The clear interest participants held for supporting other women also reflects longstanding Inuit traditions of relational knowledge-sharing (women as resources for women). This tradition has been articulated by Pauktuutit Inuit Women of Canada: “when emotionally stressed, Inuit need Inuit, their knowledge and familiarity,” underscoring the central role Inuit women may play for each other as sources of support during times of transition and change, such as the perinatal period (p. 13, [85]). For many Indigenous women, and Inuit women in particular, the perinatal period can also carry additional significance as a moment that links ancestors, present relations, and future generations through the act of bringing new life into the community [83,86,87]. The findings of this study indicate an interest in intergenerational knowledge transmission and collective care. Closely tied to the importance of learning from Elders, participants expressed a desire for knowledge in topic areas that were notably once embedded in everyday life prior to the imposition of evacuation policies (e.g., pregnancy loss, breastfeeding, nutrition, etc.). These understandings are supported by a broader literature base concerning experiential learning (learning by doing, observing, and being a part of), which offers a well-established framework that can be applied to perinatal support within Indigenous contexts [88,89]. Evacuation policies have disrupted relational roles and responsibilities; however, the findings suggest Inuit women continue to value perinatal supports that honour collective forms of care. The perinatal period, therefore, functions not only as a time of physical and emotional transition, but also as a critical moment of cultural reaffirmation for women, families, and communities [83]. The concept of Inunnguiniq, the making of a human being, is known to support the social, cultural, and spiritual dimensions of raising children within Inuit communities [46,84]. Inunnguiniq highlights how children are shaped through relationships with parents, Elders, extended kin, and community members, and how caregiving serves as a critical site for the transmission of values, identity, and cultural knowledge across generations. Within this context, SRHR can be better understood in ways that recognize and support community roles, rights, and responsibilities in care.

### 8.3. Structural Barriers and Inequities

While practices of women supporting women are deeply rooted in Inuit cultural traditions, they are also shaped by broader social and structural conditions. Existing literature has documented how the maternal evacuation policy imposes multiple, intersecting challenges and barriers to Inuit women. This includes family separation, emotional distress, financial strain, and uncertainty regarding escort eligibility during travel, among other things [16,39,42,43,44]. Differences observed between Arviat and Kangiqłiniq, despite their relatively similar size, underscore how these challenges and barriers can be experienced unevenly, pointing to the necessity of community-specific approaches to supporting women and families. Our survey findings extend this body of work by identifying gaps not only in the information provided to women, but in the specific information they identify as necessary. These findings further illuminate the structural inequities embedded within standardized, one-size-fits-all approaches to perinatal healthcare.

Although the evacuation policy produces a wide range of challenges and barriers for women in the Kivalliq region, this study focuses specifically on information gaps as a form of structural inequity (economic, physical, and structural constraints impacting Inuit in Nunavut, such as unreliable infrastructure, weather, and cost, can all limit Internet access and further restrict engagement with information offered online as well. Even when access to the Internet is available, limited English and/or digital literacy skills can make navigating web-based health information challenging, and at times, not possible at all). Participants consistently identified needs for what should be considered basic perinatal information. Participants indicated a need for information that aligned with their lived experiences, particularly regarding medical travel to Winnipeg. This is striking given that travel for birth is required (and has been for decades), and territorial health services provide information to families to support this experience. The Government of Nunavut’s Department of Health published a Nunavut Medical Travel Handbook in 2025 specifically for this purpose. The fact that so many women reported a desire for more information about travelling to Winnipeg prior to delivery signals a failure in information provision, transmission, and/or communication processes within the territory. For example, access to this information may be complicated by multiple intersecting barriers, including language and access to internet/online information. Yet this occurs in a context that has long been aware of the need for linguistically relevant and accessible health resources [90,91].

While more research is needed to determine nuanced understandings concerning participants’ specific needs for medical travel information, considering these findings in conjunction with other survey results highlights several important gaps. For example, when this desire for information is understood alongside the long-standing lack of linguistically relevant information, it may help explain women’s reliance on one another for support and knowledge. In the absence of information in their first language, informal and relational knowledge-sharing networks may be more accessible for some Inuit women than written or digital resources and, in some cases, may be the only accessible and viable source of information. This suggests that standardized, primarily English-language online perinatal resources are insufficient for Inuit communities. Such approaches risk perpetuating health inequities by failing to account for linguistic diversity, literacy, cultural context, and unequal access to resources. As a result, experiential knowledge and relational forms of learning are not only culturally meaningful sources of support but also practical and essential pathways to more equitable perinatal care.

## 9. Limitations

Since demographic information was not required by participants, the exact age distribution of all participants in this study remains unknown. Older participants were asked to reflect on observations within their families and communities, including the experiences of children, grandchildren, and others. However, it is possible that this framing introduced some ambiguity in responses, particularly in distinguishing between personal and observed experiences. Future research would benefit from the collection of more detailed demographic information and the use of a more expansive survey instrument to support deeper and nuanced analysis. Additionally, there may have been unintended ambiguity in how key concepts were interpreted. For example, healing after birth (recovery) was intended to capture primarily physical aspects of the postpartum period, while postpartum wellness was meant to reflect emotional, spiritual, and cultural dimensions of health. During analysis, however, it became evident that these distinctions may not have translated equivalently across dialects, and as a result, the participants in the two communities may have interpreted these items differently. Additional research that better articulates and explores the differences in understanding of these concepts is needed.

## 10. Conclusions

Overall, our findings reinforce understandings that birth evacuation is not only a clinical intervention but a structural process with implications for perinatal experiences, reproductive autonomy, and Inuit self-determination. By prioritizing biomedical risk management over relational and community-based approaches to care, the current system remains misaligned with Inuit needs and priorities for perinatal information, supports, and services concerning birthing and, as a result, constrains participation in decision-making.

Participants emphasized the importance of care that is grounded in Inuit knowledge, collective care and responsibility, community connection, and the guidance of Elders. These priorities provide an important foundation for revisioning perinatal health policy and practice in Nunavut. Advancing Inuit-led models of perinatal care, such as traditional Inuit midwifery, and other culturally grounded education, supports, and approaches that centre Inuit authority and reproductive autonomy, may improve experiences of care while promoting health equity and positive outcomes, cultural safety, and the right of Inuit families to shape childbirth on their own terms.

## Data Availability

The datasets presented in this article are not readily available because they are only under the stewardship of Dr. Johnston at the University of Calgary until they can be transferred to a permanent Inuit-controlled database within Nunavut. Requests to access the datasets should be directed to Dr. Johnston, and she will bring requests to the Governing Council of *Keeping the Children Home*.
